# Trypanosomatid Infections among Vertebrates of Chile: A Systematic Review

**DOI:** 10.3390/pathogens9080661

**Published:** 2020-08-16

**Authors:** Juana P. Correa, Antonella Bacigalupo, Esteban Yefi-Quinteros, Gemma Rojo, Aldo Solari, Pedro E. Cattan, Carezza Botto-Mahan

**Affiliations:** 1 Facultad de Medicina Veterinaria, Universidad San Sebastián, Concepción 4080871, Chile; juana.correa@uss.cl; 2 Institute of Biodiversity, Animal Health & Comparative Medicine, University of Glasgow, Glasgow G12 8QQ, UK; abacigalupo@uchile.cl; 3 Departamento de Ciencias Biológicas Animales, Facultad de Ciencias Veterinarias y Pecuarias, Universidad de Chile, Santiago 8820808, Chile; estebanyefi@gmail.com (E.Y.-Q.); pcattan@uchile.cl (P.E.C.); 4 Programa de Biología Celular y Molecular, Instituto de Ciencias Biomédicas, Facultad de Medicina, Universidad de Chile, Santiago 8380000, Chile; gemma.rojo@uoh.cl (G.R.); asolari@med.uchile.cl (A.S.); 5 Instituto de Ciencias Agroalimentarias, Animales y Ambientales (ICA3), Universidad de O’Higgins, San Fernando 3070000, Chile; 6 Departamento de Ciencias Ecológicas, Facultad de Ciencias, Universidad de Chile, Santiago 7800003, Chile

**Keywords:** native mammals, exotic mammals, domestic mammals, *Trypanosoma cruzi*, hosts, reservoirs, Chagas disease, *Mepraia*, *Triatoma infestans*, vector-borne parasite

## Abstract

We present a review on the natural infection by trypanosomatids of nonhuman vertebrates in Chile, aiming to synthesize and update the knowledge on the diversity of trypanosomatids infecting native and alien vertebrate species. To this end, we conducted a systematic review of literature records published from 1900 to April 2020 on four databases, focusing on the 21 genera of trypanosomatids and Chile. The methods and findings of our review have been based on the preferred reporting items for systematic reviews and meta-analysis (prisma) checklist. We found 29,756 records but only 71 presented relevant information for this review. Overall, there are only two reported trypanosomatid genera infecting vertebrate species in Chile, the genera *Trypanosoma* and *Leishmania*. The former is mostly represented by *Trypanosoma cruzi* (90% of the total records) and to a much lesser extent by *Trypanosoma avium*, *Trypanosoma humboldti*, *Trypanosoma lewisi*, and a couple of unidentified trypanosomatids. A total of 25 mammals have been reported as being infected by *T. cruzi*, including 14 native and 11 alien species from Orders Artiodactyla, Carnivora, Chiroptera, Didelphimorphia, Lagomorpha, Perissodactyla, and Rodentia. Extensive screening studies using new analytical tools are necessary to grasp the whole potential diversity of trypanosomatid species infecting vertebrates in Chile.

## 1. Introduction

Trypanosomatidae corresponds to a diverse family of protozoan parasites of the class Kinetoplastea, whose development is predominantly restricted to a single host species. However, some trypanosomatids can use more than one host species throughout its life cycle. The Trypanosomatidae family includes 21 genera parasitizing invertebrate, vertebrate and/or plant species [1,2]. Several species of the genus *Leishmania* and *Trypanosoma* play important roles as human pathogens, causing several infectious diseases in which insect vectors are involved in their transmission [3]. Some of the most relevant vector borne infectious diseases in America are leishmaniasis (caused by several *Leishmania* species) and American trypanosomiasis (or Chagas disease, caused by *Trypanosoma cruzi*), both considered neglected tropical diseases mainly affecting poor people from the low-income countries of Central and South America [3,4]. 

Chile is a South American country considered a biogeographic island due to the presence of the extremely arid desert in the north, the Antarctic waters in the south, the Andes Range in the east, and the Pacific Ocean in the west. The main biomes present in continental Chile are (i) deserts and xeric shrublands, (ii) Mediterranean forests, woodlands and scrub, (iii) montane grasslands and shrublands, and (iv) temperate broadleaf and mixed forests [5]. These geographic features partially explain the low species richness and high levels of endemism found in the flora and fauna of this area [6]. Terrestrial vertebrates are not the exception, with amphibians, reptiles, and mammals exhibiting 65, 63, and 11% of endemic species, respectively [6]. In spite of this interesting feature potentially leading to endemic host–parasite interactions, little is known about the trypanosomatids infecting native vertebrate species in this country. In addition, the introduction of alien animals (livestock, game, pet, and synanthropic species) since the XVI century [7], carrying their own parasitic fauna from their original native regions [8], makes the study of parasites even more relevant.

In Chile, the most studied trypanosomatid is *Trypanosoma cruzi*, transmitted by four triatomine vector species: the mainly domiciliated species *Triatoma infestans*, and the three wild endemic species *Mepraia gajardoi*, *Mepraia parapatrica* and *Mepraia spinolai* [9,10,11,12,13,14]. This flagellated protozoan has been reported infecting native as well as alien mammals [14,15,16,17]. However, the published reports on *T. cruzi* infection in mammals are scattered, without an exhaustive, organized and unbiased review of the available information for this as well as for other trypanosomatids potentially present in Chile.

The aim of this review is to synthesize and update the knowledge on the diversity of trypanosomatids infecting nonhuman vertebrates in Chile, including native and alien vertebrate host species. To this end, we conducted a systematic review of literature records published from 1900 to April 2020, focusing on the 21 genera of trypanosomatids described [1], based on the preferred reporting items for systematic reviews and meta-analysis (PRISMA) checklist.

## 2. Results

**Figure 1:**
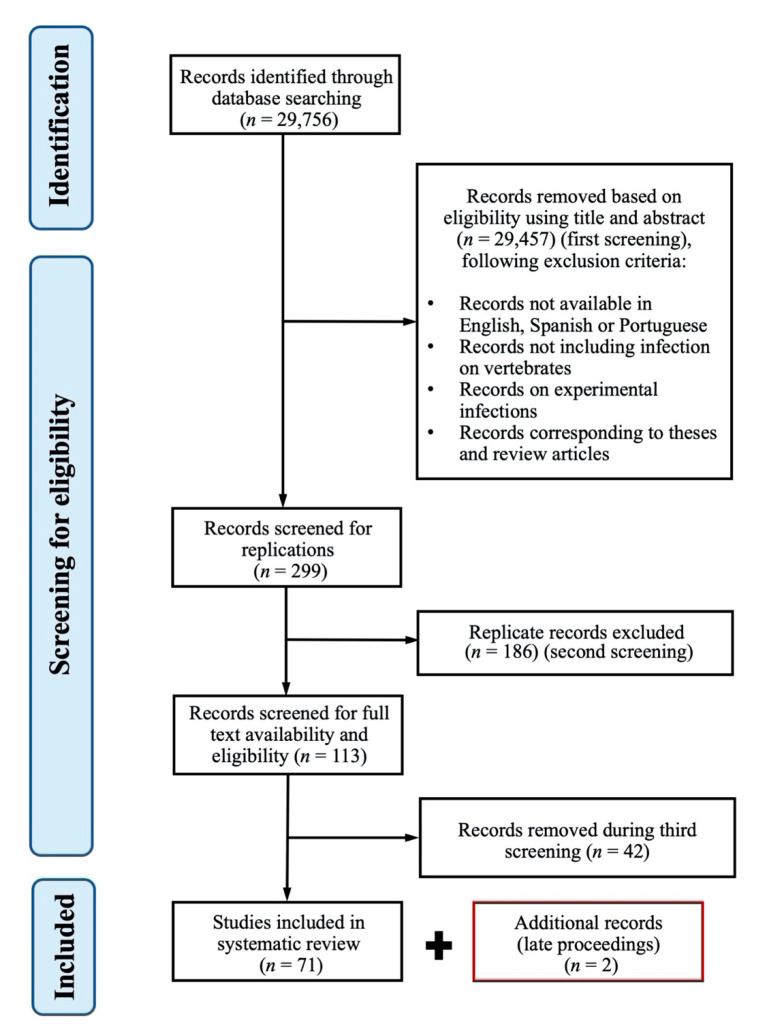
PRISMA (preferred reporting items for systematic review and meta-analysis) flowchart diagram of the record selection process. Additional records included in this review are depicted in a red box.

In total, 29,756 records were obtained before the screening process. After the first screening the literature search identified a total of 299 records: 101 by Google Scholar (1921–2020), 87 by Web of Science (1915–2020), 57 by EMBASE (1949–2020), and 54 by PubMed (1952–2020). One-hundred and eighty-six replicated records were removed after the second screening. A substantial number of the articles thus obtained were subsequently removed mostly after reading the full texts (n = 41) or when the full texts were unavailable (n = 1). A total of 71 articles was retained for this systematic literature review and two additional records were added (Figure 1). These two were abstracts published in a scientific journal after the closure of our search, presenting new infected vertebrate species in Chile.

We only found and maintained the records of two trypanosomatid genera: *Leishmania* (n = 2 records) and *Trypanosoma* (n = 71 records), and one undetermined trypanosomatid genus (n = 1 record). One record reported both *Leishmania* and *Trypanosoma*. For the genus *Leishmania*, the presence of *Leishmania* spp. was tested in the endemic Darwin’s fox *Lycalopex fulvipes* by means of the quantitative polymerase chain reaction (qPCR, herafter) without positive detection [18], but a recent study using the same technique detected *Leishmania* sp. in *Canis lupus familiaris* [19]. 

For the genus *Trypanosoma*, at least four species were recorded infecting vertebrates: *T. avium*, *T. cruzi, T. humboldti*, and *T. lewisi*. In addition, one unidentified *Trypanosoma* sp. was detected by means of optical microscopy in the passerine *Phrygilus fruticeti* [20] and one undetermined trypanosomatid was reported in the camelid *Lama guanicoe* by conventional PCR (cPCR, hereafter) [21] (Table 1). Another report searched for *Trypanosoma* spp. in several bird species from Northern Chile by molecular methods without positive detection [22].

**Figure 2:**
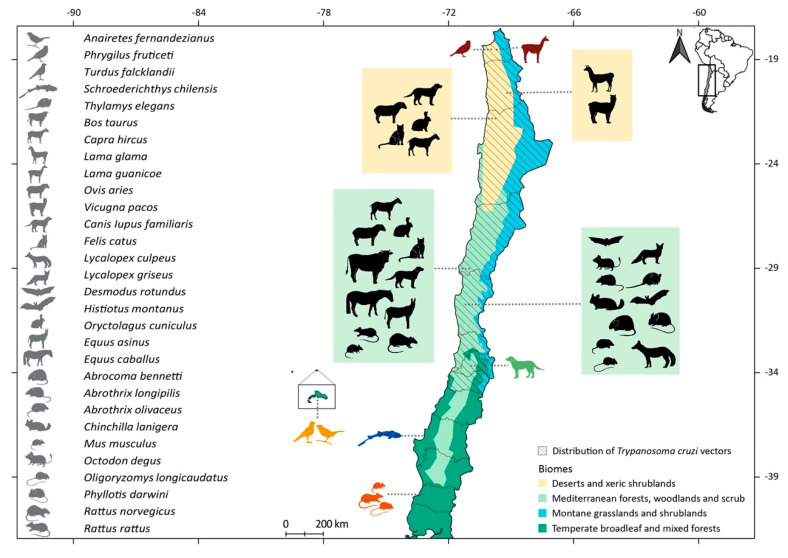
Map of Chile depicting the geographic location and the biomes used by triatomines and vertebrate species infected by trypanosomatids, and a reference map showing the location of Chile in South America. Colors in the vertebrate species indicate infection by *Leishmania* (green), *Trypanosoma avium* (dark yellow), *Trypanosoma cruzi* (black), *Trypanosoma humboldti* (blue), *Trypanosoma lewisi* (red), unidentified *Trypanosoma* sp. or trypanosomatid (brown). On the left, species scientific names are indicated. Native and alien vertebrate species infected by *T. cruzi* are shown to the right and left of Chile, respectively, in colored boxes representing the color of the biome where these infected vertebrates were detected. For the rest of the trypanosomatid-infected species, the exact locations are shown. The distribution of triatomine species includes *Mepraia gajardoi*, *M. parapatrica*, *M. spinolai*, and *Triatoma infestans*. Division lines inside the Chilean territory represent the administrative regions from North to South: Arica y Parinacota, Tarapacá, Antofagasta, Atacama, Coquimbo, Valparaíso, Metropolitana, O’Higgins, Maule, Biobío, Araucanía, Los Ríos, and Los Lagos. The zoom on the Pacific Ocean corresponds to Robinson Crusoe island from the Juan Fernández Archipelago.

**Figure 3:**
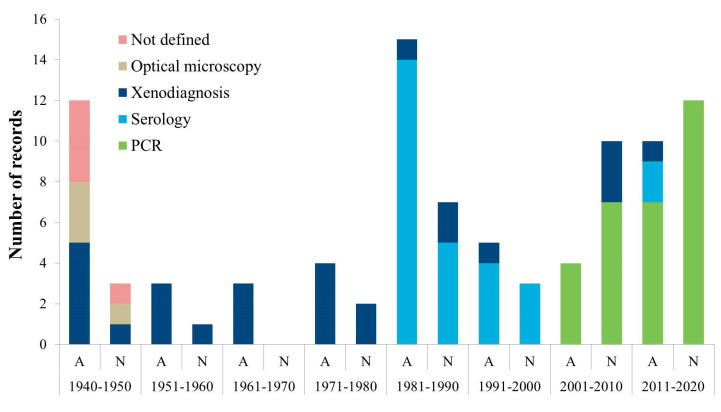
Number of records per decade published on *Trypanosoma cruzi* infection according to diagnostic techniques on alien (A) and native (N) mammals from Chile. When a record presented two diagnostic techniques, this record was assigned to both techniques, the same as when it presented both native and alien species. Techniques: optical microscopy on blood; xenodiagnosis; serology including IHA (indirect hemagglutination), IIF (indirect immunofluorescence), and ELISA (enzyme-linked immunosorbent assay); PCR (polymerase chain reaction), including cPCR (conventional PCR), cPCR+SB (cPCR and Southern blot), hnPCR (hemi-nested PCR), nPCR (nested PCR), real-time PCR, and qPCR (quantitative PCR).

Within the reported *Trypanosoma* spp. included in our selection, *T. avium* has been detected by PCR and sequencing in the passerines *Anairetes fernandezianus* and *Turdus falcklandii* from the Robinson Crusoe island [23]; *T. humboldti* was detected by optical microscopy in the redspotted catshark *Schroederichthys chilensis* [24,25,26]; *T. lewisi* was detected by optical microscopy in the rodents *Mus musculus*, *Oligoryzomys longicaudatus*, and *Rattus rattus* [27]; *T. cruzi* has been tested in 41 mammal species and recorded infecting 25 species from seven Orders, which are detailed below. See the geographic location and the biomes where vertebrates infected by trypanosomatids were detected in Figure 2, the temporal distribution of *T. cruzi* records in Figure 3, and the summaries of the native and alien mammals infected by *T. cruzi* in Table 2 and Table 3, respectively.

### 2.1. Native Mammal Hosts

#### 2.1.1. Order Didelphimorphia

In Chile, two species of marsupials of the Order Didelphimorphia have been described [28]. The distribution of the endemic insectivorous species, *Thylamys elegans*, overlaps with the geographic distribution of *M. spinolai* and *T. infestans* [10,12,13,28]. Studies of *T. cruzi* infection in *T. elegans* have shown a high variability, from the complete absence of infection when assessed by optical microscopy, serology (indirect hemagglutination; IHA, hereafter), and/or xenodiagnosis (XD, hereafter) [29,30], to a range of 28.6–50.0% of infection by cPCR on blood [14,17,31,32,33,34]. See the detailed information in Table 2 and Supplementary Materials. The *T. cruzi* discrete typing units (DTUs, hereafter) [35] reported circulating in *T. elegans* are TcI, TcII, TcV, and TcVI [14,31].

#### 2.1.2. Order Artiodactyla

In Chile, seven species of even-toed ungulates have been described, including camelids and deers [28]. Five of these species might overlap with the geographic distribution of the *Mepraia* species and *T. infestans* [10,12,13,28], and two of them are domesticated camelids (*Lama glama* and *Vicugna pacos*) closely related to people from rural areas [28]. Serological studies of *T. cruzi* infection in the camelids *L. glama* and *V. pacos* have shown evidence of infection. In the former, some studies have reported between 0 and 0.7% of infection [36,37,38,39], while in the latter between 1.4 and 11.2% of infection have been detected [16,38]. See the detailed information in Table 2 and Table S1 in Supplementary Materials.

#### 2.1.3. Order Carnivora

In Chile, 25 species of carnivores have been described [28]. Several of these carnivores, including felids (e.g., mountain lion), canids (e.g., foxes), mustelids (e.g., lesser grison, sea otter), otariids (e.g., seals), and mephitids (e.g., skunks) overlap with the geographic distribution of the *Mepraia* species and *T. infestans* [10,12,13,28]. Among them, only three species have been tested for *T. cruzi* infection: the foxes *Lycalopex culpaeus* and *Lycalopex griseus* [15,29,40,41,42,43,44] and the skunk *Conepatus chinga* [29]. Only the foxes have been detected infected with *T. cruzi*, ranging from 0 to 1.3% of infection prevalence in *L. culpaeus* and 0 to 3.8% in *L. griseus* using optical microscopy, serology (IHA) and/or XD [15,29,40,41,42,43,44]. See detailed information in Table 2 and Table S1 in Supplementary Materials.

#### 2.1.4. Order Chiroptera

In Chile, 11 species of bats have been described [28]. Several of these insectivorous or hematophagous species overlap with the geographic distribution of the *Mepraia* species and *T. infestans* [10,12,13,28]. Studies of *T. cruzi* infection in bat species are scarce. The species *Histiotus macrotus*, *Lasiurus borealis*, *Lasiurus cinereus*, *Myotis chiloensis* and *Tadarida brasiliensis* were tested in the 1940s for *T. cruzi* infection by optical microscopy and XD but no infection was detected [29]. However, a recent preliminary study [45], carried out in two protected areas in the northern region of Chile, detected the presence of *T. cruzi* DNA by real-time PCR. The study evaluated different types of biological samples from *Desmodus rotundus*, *Histiotus montanus*, *M. chiloensis*, and *Histiotus* sp. and pools of feces collected in bat roosts to test the presence of *T. cruzi*. Both *H. montanus* and *D. rotundus* were positive to *T. cruzi* DNA. However, according to the authors, the *T. cruzi* transmission mechanism is still unknown (oral, congenital or vectorial) due to the insectivorous and hematophagous feeding habits of *H. montanus* and *D. rotundus*, respectively. See the detailed information in Table 2 and Table S1 in Supplementary Materials.

#### 2.1.5. Order Rodentia

In Chile, 68 species of rodents have been described [28]. More than half of these species overlap with the geographic distribution of the *Mepraia* species and *T. infestans* [10,12,13,28]. Among them, 13 species have been tested for *T. cruzi* infection, including three *Abrothrix* species, two *Octodon* species, *Abrocoma bennetti*, *Chinchilla lanigera*, *Lagidium viscacia*, *O. longicaudatus*, *Phyllotis darwini*, and *Spalacopus cyanus*, among others. From these, seven species have been reported as being infected by *T. cruzi*, with a high variation in the frequency of infection, mainly depending on the detection technique: *A. bennetti* (0–42.9%), *Abrothrix longipilis* (0–9.5%), *Abrothrix olivaceus* (0–71.0%), *C. lanigera* (20.0–40.0%), *Octodon degus* (8.3–70.4%), *O. longicaudatus* (0–50.0%), and *P. darwini* (0–100%) [14,15,17,29,30,31,32,33,34,40,41,42,43,44,46,47,48,49,50,51,52,53,54,55,56,57]. See the detailed information in Table 2 and Table S1 in Supplementary Materials. The *T. cruzi* DTUs reported as circulating in *A. olivaceus*, *O. degus*, and *P. darwini* are TcI, TcII, TcV, and TcVI; in *O. longicaudatus*, TcI, TcV and TcVI; in *A. longipilis* only TcI was reported [14,31,47,48,49].

### 2.2. Alien Mammal Hosts

#### 2.2.1. Order Artiodactyla

Eight species of even-toed ungulates have been introduced in Chile, including four deer species, cattle (*Bos taurus*), and caprine (*Capra hircus*), ovine (*Ovis* spp.) and porcine (*Sus scrofa*) livestock [28]. Free-ranging individuals of these species can also be found in several ecosystems of Chile [28]. Six of these species might overlap with the geographic distribution of the *Mepraia* species and/or *T. infestans* [10,12,13,28]. Among them, four species have been tested for *T. cruzi* infection (*B. taurus*, *C. hircus*, *Ovis aries*, and *S. scrofa*), with infections reported in the first three. Only few studies have assessed *T. cruzi* infection in *B. taurus* and *O. aries*, showing a high variability depending on the diagnostic technique. Only serological studies (IHA) carried out in the 1980s reported that some specimens of these species had been exposed to *T. cruzi* infection: up to 13.4 and 18.8% in *B. taurus* and *O. aries*, respectively [16,36,37,39,42,58,59,60,61,62,63,64,65,66]. Studies of *T. cruzi* infection in *C. hircus* have shown a high variability, from 0 to 35.0% when assessed by optical microscopy, serology (IHA and indirect immunofluorescence; IIF, hereafter), and/or XD [15,16,29,36,37,39,42,43,59,60,61,62,63,64,65,67,68,69,70,71], to 31.0 and 50.0% of infection when assessed by XD coupled with cPCR, and cPCR on blood, respectively [17,31,72]. See the detailed information in Table 3 and Table S2 in Supplementary Materials. Only one study has reported the *T. cruzi* DTUs circulating in *C. hircus* of an endemic area, detecting TcI, TcII, TcV, and TcVI [31].

#### 2.2.2. Order Carnivora

Four species of carnivores have been introduced in Chile [28]. Two of these, *Canis lupus familiaris* and *Felis catus*, were introduced in the XVI century as pets, but free-ranging specimens of both species can be found in several ecosystems [28]. Domestic and free-ranging individuals of both types of carnivore might overlap with the geographic distribution of the *Mepraia* species and/or *T. infestans* [10,12,13,28]. Several studies have assessed *T. cruzi* infection in *C. l. familiaris* and *F. catus*, which have shown a high variability, mainly depending on the diagnostic technique and, to a lesser extent, on the location of the populations prospected. The infection prevalence in *C. l. familiaris* ranges from 0 to 34.8% when assessed by optical microscopy, XD, and serology (IHA, IIF and enzyme-linked immunosorbent assay; ELISA) [15,16,29,36,37,39,40,41,42,43,58,59,60,61,62,63,64,65,66,67,68,70,73,74,75,76,77,78,79,80,81,82,83,84,85], while few studies using cPCR, real-time PCR and nested PCR showed infection prevalence from 17.1 to 35.2% [19,86,87]. The infection prevalence in *F. catus* ranges from 0 to 23.4% when assessed by optical microscopy, XD, and serology (IHA) [15,16,29,36,37,39,40,41,42,43,58,59,60,61,62,63,64,65,67,68,73,74,75,76,77,78,79,80,81,82,83]. See the detailed information in Table 3 and Table S2 in Supplementary Materials. Only one study has reported the *T. cruzi* DTUs circulating in *C. l. familiaris* of several localities from endemic areas, detecting TcI, TcII, TcIII, TcV, and TcVI [87].

#### 2.2.3. Order Lagomorpha

Two species of lagomorphs were introduced in Chile by the end of the XIX century, the hare *Lepus europaeus* and the rabbit *Oryctolagus cuniculus* [28]. Both free-ranging species overlap with the geographic distribution of *M. spinolai* and *T. infestans* [10,12,13,28]. Several studies have assessed *T. cruzi* infection in *O. cuniculus*, which have shown some variability depending on the location of the populations prospected and the diagnostic technique used. In early studies, the infection prevalence ranged from 0 to 12.1% when studied using optical microscopy, serology, and XD [15,16,29,36,37,39,40,41,42,43,59,60,61,62,63,64,65,67,83], while a study using cPCR and hemi-nested PCR showed an infection prevalence of 19.0% and 37.9%, respectively [88]. Only one study carried out in the 1940s, with a very small sample size, assessed *T. cruzi* infection by optical microscopy and XD in *L. europaeus*, and no infection was detected [29]. See the detailed information in Table 3 and Table S2 in Supplementary Materials. The *T. cruzi* DTUs reported circulating in *O. cuniculus* are TcI, TcII, TcV, and TcVI [88]. 

#### 2.2.4. Order Perissodactyla

Two species of odd-toed ungulates were introduced in Chile by the end of the XVI century or later, the ass *Equus asinus* and the horse *Equus caballus* [28]. Both species overlap with the geographic distribution of *M. spinolai* and *T. infestans* [10,12,13,28], and even though they are mainly domesticated species, some free-ranging populations can be found [28]. Several studies have assessed *T. cruzi* infection in both species using serology or XD. In *E. asinus* and *E. caballus*, the infection prevalence ranges between 0 and 17.8% and 0 and 15.8%, respectively [16,29,37,39,42,60,61,62,63,65,66,67]. See the detailed information in Table 3 and Table S2 in Supplementary Materials.

#### 2.2.5. Order Rodentia 

Six species of rodents have been accidentally or intentionally introduced into Chile, including several rat species, the common house mouse, and the beaver [28]. Three of these species (*M. musculus*, *Rattus norvegicus*, and *R. rattus*) overlap with the geographic distribution of *Mepraia* species and *T. infestans* [10,12,13,28]. Few studies have assessed *T. cruzi* infection in alien rodent species, which have shown a high variability depending on the species, location of the population prospected, and the diagnostic technique used. In the 1940s, the prevalence of *T. cruzi* infection in *R. rattus*, *R. norvegicus* and *M. musculus* was tested by optical microscopy and/or XD but no infection was detected [29]. Recent studies, using molecular detection (cPCR, hemi-nested PCR, and/or qPCR), have reported a high infection prevalence: *R. rattus* (27.7%, 83.6%), *R. norvegicus* (71.4%), and *M. musculus* (83.3%) [14,34,48,56]. See the detailed information in Table 3 and Table S2 in Supplementary Materials. Even though four DTUs, TcI, TcII, TcV and TcVI, have been tested in *R. rattus* and *R. norvegicus*, only in the former species were the four DTUs detected [14,48]. The domestic rodent *Cavia porcellus* is not included in the count of introduced rodents; however, this species has been used as food source, and it was tested for *T. cruzi* infection using XD and serology, with negative results [16,39,40,41,42,60,63,65,68]. See the detailed information in Table S2 in Supplementary Materials.

Besides the records filtered by our systematic review, *Trypanosoma equiperdum*, *Trypanosoma evansi*, and *Trypanosoma vivax* were briefly mentioned as being present in Chile [89,90], but these records did not provide further details of the findings. On the other hand, *Trypanosoma rangeli* was reported once in triatomine bugs [91]. Due to the lack of information and/or no report of infection in vertebrates, these were not included in the selected results. The complete dataset with the information obtained from each selected record used in this review can be found in Table S3 in Supplementary Materials.

## 3. Discussion

In this systematic review, we detected two genera of trypanosomatids reported in the vertebrates of Chile: *Leishmania* and *Trypanosoma*. Species of these genera have been detected in the mainland, in one island, and in the Pacific Ocean. *Leishmania* spp. has been detected only in dogs (~33°26′ S, 70°39′ W) from the Mediterranean forests, woodlands and scrub biome [19]. *Trypanosoma avium* has only been detected in two bird species from the Juan Fernández Archipelago (~33°37′ S, 78°50′ W) [23]; *T. cruzi* has been found in 14 native and 11 alien mammal species from two biomes—the deserts and xeric shrublands, and the Mediterranean forests, woodlands and scrub (~17°30′-34°36′ S, 68°12′-71°50′ W) (see Table 2 and Table 3); *T. humboldti* was detected in one small shark species from the Pacific Ocean (~36°36′ S, 72°84′ W) [24,25,26]; *T. lewisi* was found in one native and two alien rodent species from the temperate forest biome (~39°50′ S, 73°13′ W) [27]. Nonetheless, other unidentified *Trypanosoma* sp. and another trypanosomatid were reported in one bird and one camelid species, respectively [20,21], as well as *T. equiperdum*, *T. evansi*, and *T. vivax*, which have been briefly mentioned as present in Chile [89,90], but without any additional information to obtain the original sources reporting these findings. In addition, it is worth mentioning that *T. rangeli* was reported in triatomine bugs [91]. At this point, we cannot discard that some of those last-mentioned *Trypanosoma* species had been misclassified when identified by morphology or as a result of a cross reaction of serological techniques. In general, describing new trypanosomatid species in Chile is difficult because only few studies search for new parasite species, and most studies tend to add to the knowledge on already described species.

In this study, we were able to gather information on the number of vertebrate species analyzed for trypanosomatid infection in Chile. Only 55 of the ~2000 vertebrate species described for the country have been tested [92], and 30 of these were found infected by at least one trypanosomatid species. This reflects the patent need to increase the number of studies on untested Chilean vertebrate taxa, by means of analytical tools that can grasp the potential diversity of trypanosomatid species. Compared to neighboring countries (Argentina, Bolivia, and Peru), Chile presents a lower diversity of *Trypanosoma* and *Leishmania* species. For example, in all the neighboring countries—besides *T. cruzi*—there are reports of *T. evansi*, and only Bolivia and Peru have records of *T. vivax* [93,94]. Argentina has both *Trypanosoma minasense* and *Trypanosoma theileri*, and *T. lewisi* was reported in Peru [95,96,97]. On the other hand, *Leishmania braziliensis* is described in Peru and Bolivia, *Leishmania* (*Viannia*) sp. in Argentina, *Leishmania amazonensis* and *Leishmania infantum* in Bolivia, and *Leishmania guyanensis* and *Leishmania peruviana* are reported in Peru [98,99]. All of these are records of natural infection in mammals, showing that the lack of research in other taxa is frequent in the Southern Cone of South America. These differences with neighboring countries may be the result of scarce, potentially biased screenings, and/or a lack of diversity in Chile. Notwithstanding, comparisons with other countries must be considered with caution, as we did not find any comprehensive review such as the one presented here.

Future studies in Chile should also focus on other potential invertebrate vectors of trypanosomatids such as leeches [100], ticks [101,102] (but see [103]), mites [104], flies [105,106], mosquitoes [107], sandflies [108], and tabanids [106], which might explain the recent finding of the *Leishmania donovani* complex in *L. griseus* from the Argentinian Patagonia without reports of sandfly vectors [109]. In fact, there are some reports of sandflies in Northern Chile [110], so the possibility of *Leishmania* spp. being introduced or even already infecting vertebrates in Chile cannot be discarded. Future research programs should join efforts from different disciplines including entomologists, mastozoologists, herpetologists, ornithologists, ichthyologists, parasitologists, veterinarians, epidemiologists, and molecular biologists, among others, to increase trypanosomatid screenings of the overlooked groups of vertebrates. This would increase our knowledge on the trypanosomatids circulating in Chile, their putative invertebrate vector species, transmission cycles, potential risks to humans and other vertebrates, and therefore, awareness to public health programs.

The exception to the scarcity of studies on trypanosomatids is *T. cruzi*, with over 90% of the selected records. In fact, 74.6% of the total number of vertebrate species reported in the records were tested for this parasite. *Trypanosoma cruzi* has been studied for almost 80 years in the vertebrates of Chile. Most of the studies were focused on alien species until the 1980s, when native species’ studies started to increase. There seems to be a recent impressive lag of 35 years with no studies on livestock—excluding goats—and domestic cats. Regarding the diagnostic techniques used, there was a temporal shift, with XD and microscopy appearing more frequently in the earlier studied decades (1940s to 1970s), changing to serology as the predominant technique in the 1980s and 1990s, and lately, since 2000, PCR has become the preferred analytical tool. Among the multiple challenges, there is an urgent need to update the information on the *T. cruzi* infection of livestock and companion animals from rural endemic areas using molecular detection techniques, encompassing the whole area where triatomine vectors can be found. In addition, new *T. cruzi* hosts could be identified by analyzing triatomine alimentary profiles using molecular techniques [111].

In Chile, the domestic transmission cycle by the vector *T. infestans* was interrupted in 1999, which means that there are no colonies established inside houses, but the intrusion of sylvatic triatomines—of the genus *Mepraia* and *T. infestans*—is still a nuisance for people living in endemic areas [10,112,113]. Sylvatic transmission to native and alien vertebrates occurs in wild habitats; transhumant livestock can transport the infection to peridomestic areas [72], and synanthropic species can also move between these environments [48,56]. Sylvatic triatomines invade dwellings, carrying *T. cruzi* that can be transmitted to vertebrates, and accidentally to humans. Besides the stercorarian transmission of this trypanosomatid, the oral route should be considered, by vector consumption and predator–prey interactions among mammals [114]. The possibility of *T. cruzi* transmission by the congenital route in the wild is a topic that has not been addressed in Chile, and it is an incipient line of research with just one report from another South American country [115].

Trypanosomatid–vertebrate interactions occur in the context of anthropic disturbances of ecosystems caused by land use change, desertification and the introduction of alien species, with habitat degradation, loss and/or fragmentation, which affects native vertebrates and vectors [7,116]. Furthermore, climate change may influence the geographic distribution range of hosts and vectors [12,13,117], which in turn could increase parasite distribution with the potential to infect new host species and/or new populations of already reported host species. Chile has not presented autochthonous *Leishmania* infection in nonhuman vertebrates until recently [19]. Climate change could further modify this scenario by facilitating the establishment of permanent populations of phlebotomine vectors [110], which, combined with the presence of a large number of free-roaming dogs [118], offers the potential of a complete cycle for this parasite. In aquatic ecosystems, contamination could also modulate the infection by trypanosomatids in fish [26].

Possible limitations of our study comprise the use of currently accepted trypanosomatid genera [1], so previous nomenclature could have been overlooked in our search. Secondly, only four databases were used to perform the systematic review; it is possible that other sources could have provided additional relevant records. We did not include these as sources, given that our access to these types of records would have been limited and probably biased. Regarding the abstracts published in the proceedings, only a few actually met our requirements of including quantifiable data on trypanosomatid infection in vertebrates, but some of the discarded records probably could have provided more information if access to their whole presentation would have been granted by the authors upon contact.

In conclusion, only two genera of trypanosomatids have been reported in the vertebrate species present in Chile, and most of the reports obtained in this systematic review corresponded to *T. cruzi*. In the future, more trypanosomatid species in Chile could be identified and described, as well as new vertebrate hosts, and/or new locations for these parasite species. These reports could correspond to discoveries of well-established cycles not previously detected, or to the introduction of new parasites, hosts or vectors. Unfortunately, advances on this matter would depend on the science budget allocated to research focused on transdisciplinary prospective long-term screening programs, searching for parasites with zoonotic potential, assessing infection in alien fauna with economic impacts, and testing infection in native species to anticipate potential biodiversity losses.

## 4. Materials and Methods 

### 4.1. Systematic Review Protocol, Search Strategy and Data Collection

The systematic literature review followed the standard systematic review procedures established by the preferred reporting items for systematic reviews and meta-analyses (PRISMA). Between January and April 2020, four scientific database search engines (EMBASE, Google Scholar, PubMed, and Web of Science) were used to identify articles and the search terms (trypanosomatid OR Blastocrithidia OR Blechomonas OR Herpetomonas OR Jaenimonas OR Lafontella OR Crithidia OR Endotrypanum OR Leishmania OR Leptomonas OR Lotmania OR Wallaceina OR Zelonia OR Novymonas OR Paratrypanosoma OR Phytomonas OR Sergeia OR Angomonas OR Kentomonas OR Strigomonas OR Trypanosoma OR Wallacemonas) AND (Chile) were included, searching records from 1900 to 2020, when available.

### 4.2. Study Selection

The records included for this literature review were retrieved through three screening phases. The first screening phase evaluated the titles and abstracts regarding the relevance to the review. Four exclusion criteria were applied on the first screening phase: (i) records such as theses and review articles (retaining articles such as short communications, full papers, preprints, and abstracts published in congress proceedings); (ii) records not available in English/Spanish/Portuguese; (iii) records not including vertebrates; (iv) records not including natural infection. A second screening removed replicates using citations and titles (including records with the same title in different languages), after merging the results obtained from the different literature sources; while the third screening phase was applied to the full texts. The main reasons for exclusion in the last screening phase was that full text was not available, and that after reading the full text the article did not comply with reporting natural infection by trypanosomatids in nonhuman vertebrates. The final selected articles were first categorized by trypanosomatid genus (e.g., *Trypanosoma* spp., *Leishmania* spp.), and we extracted the vertebrate species, the time frame of the study, the region/province/locality (if available), coordinates (if any), the diagnostic method, type of biological sample, number of individuals tested, number of individuals infected, and the frequency of infection. In the absence of a detailed database in the electronic supplementary material of recently published articles, the corresponding authors were contacted to obtain additional information. Records from each category were organized by host class (e.g., Amphibia, Aves, Mammalia, Reptilia), followed by the Order, host species and diagnostic methodology.

Figures were prepared using PowerPoint and Excel for Mac (version 16.35), and QGIS 3.10.1 (http://qgis.osgeo.org), with basemaps for Chile (http://labgeo.ufro.cl), South America (https://tapiquen-sig.jimdofree.com), and biomes (https://ecoregions2017.appspot.com).

## Figures and Tables

**Table 1:** Summary of the trypanosomatids infecting vertebrates in Chile.

Trypanosomatid Species	Host Class	Species
*Leishmania* sp.	Mammalia	*Canis lupus familiaris*
*Trypanosoma avium*	Aves	*Anairetes fernandezianus, Turdus falcklandii*
*Trypanosoma cruzi*	Mammalia	*Abrocoma bennetti*, *Abrothrix longipilis*, *Abrothrix olivaceus*, *Bos taurus*, *Canis lupus familiaris*, *Capra hircus*, *Chinchilla lanigera*, *Desmodus rotundus*, *Equus asinus*, *Equus caballus*, *Felis catus, Histiotus montanus*, *Lama glama*, *Lycalopes culpaeus*, *Lycalopex griseus*, *Mus musculus, Octodon degus*, *Oligoryzomys longicaudatus*, *Oryctolagus cuniculus*, *Ovis aries*, *Phyllotis darwini*, *Rattus norvegicus*, *Rattus rattus*, *Thylamys elegans, Vicugna pacos*
*Trypanosoma humboldti*	Carcharhiniformes	*Schroederichthys chilensis*
*Trypanosoma lewisi*	Mammalia	*Mus musculus, Oligoryzomys longicaudatus, Rattus rattus*
*Trypanosoma* sp.	Aves	*Phrygilus fruticeti*
Trypanosomatid	Mammalia	*Lama guanicoe*

**Table 2:** Native mammals infected by *Trypanosoma cruzi* in Chile.

ORDER/Species	Region(s)	Positive/ Total Tested (%)	Assay Type (Sample Type)	Reference
DIDELPHIMORPHIA				
Elegant fat-tailed opossum	AT/CO/VA/ME	0/15	OM, XD	[29]
(*Thylamys elegans*)	CO	0/4	XD, IHA	[30]
	CO	6/13 (46.2)	cPCR+SB (blood)	[17,31]
	CO	8/28 (28.6) ^a^	cPCR (blood)	[32]
	CO	2/4 (50.0) ^a^	cPCR (blood)	[33]
	CO/VA/ME	6/14 (42.9) ^b^	cPCR (blood)	[14,34]
ARTIODACTYLA				
Llama	AP/TA	0/182	IHA	[36,49]
(*Lama glama*)^c^	AN	0/37	IHA	[37,39]
	AP	1/136 (0.7)	IHA	[38]
Alpaca	AP	49/439 (11.2)	IHA	[16]
(*Vicugna pacos*)^c^	AP	29/2011 (1.4)	IHA	[38]
CARNIVORA				
Culpeo fox	AT/VA/ME	7/533 (1.3) ^b^	OM, XD	[15,29,40,41,42,43]
(*Lycalopex culpaeus*)	CO	0/1	IHA	[44]
South American gray fox	AP/TA/AN/AT/VA/ME/OH	3/78 (3.8) ^b^	XD	[15,29,40,41,42,43]
(*Lycalopex griseus*)	CO	0/2	IHA	[44]
CHIROPTERA				
Common vampire bat (*Desmodus rotundus*)	AT	6/17 (35.3)	Real-time PCR (tissue)	[45]
Small big-eared brown bat (*Histiotus montanus)*	CO	4/8 (50.0)	Real-time PCR (anal swab/feces)	[45]
RODENTIA				
Bennett’s chinchilla-rat	AT/VA/ME	0/43	OM, XD	[29]
(*Abrocoma bennetti*)	CO	4/11 (36.4)	XD, IHA	[30]
	ME	0/2	hnPCR (blood)	[48]
	CO	3/7 (42.9)	cPCR (blood)	[51]
	CO	4/12 (33.3)^a^	cPCR (blood)	[33]
	CO	2/9 (22.2)	cPCR (blood)	[14,34]
Long-haired grass mouse	NK	0/2	OM	[29]
(*Abrothrix longipilis*)	CO	0/1	XD, IHA	[30]
	CO	0/1	IHA	[44]
	CO/ME	2/21 (9.5)	cPCR (blood)	[14,34]
Olive grass mouse	AT	0/5	OM	[29]
(*Abrothrix olivaceus*)	CO	0/4	IHA	[30]
	CO	31/44 (71.0)	cPCR+SB (blood)	[17,31]
	ME	0/2	hnPCR (blood)	[48]
	CO	20/32 (62.5)	cPCR (blood)	[51]
	CO	36/89 (40.5) ^a^	cPCR (blood)	[33]
	CO/VA/ME	16/41 (39.0)	cPCR (blood)	[14,34]
	CO	15/45 (33.3) ^a^	cPCR (blood)	[57]
Long-tailed chinchilla	CO	7/35 (20.0)	XD, IHA	[30]
(*Chinchilla lanigera*)	CO	8/20 (40.0)	XD, IHA	[44]
Degu	AT/CO/ME	9/412 (2.2) ^b^	OM, XD, IHA	[15,29,40,41,42,43]
(*Octodon degus*)	CO	5/60 (8.3)	XD, IHA	[30]
	CO	3/14 (21.4)	IHA	[44]
	CO	28/46 (61.0)	cPCR+SB (blood)	[17,31]
	CO	8/35 (22.9) ^a^	XD-cPCR	[46,47,49]
	ME	8/60 (13.3)	hnPCR (blood)	[48]
	CO	38/96 (39.6)	cPCR (blood)	[32]
	CO	68/140 (48.6)	cPCR (blood)	[50]
	CO	69/98 (70.4)	cPCR (blood)	[51]
	CO	106/262 (40.5)	cPCR (blood)	[53,54]
	CO	170/460 (37.0) ^ab^	cPCR (blood)	[33,52]
	CO	40/57 (70.2)	cPCR (blood)	[55]
	CO	2/4 (50.0)	qPCR (blood)	[56]
	CO	107/273 (40.2) ^a^	cPCR (blood)	[57]
Degu and moon-toothed degu (*O. degus* and *O. lunatus*)	CO/VA/ME	89/356 (25.0)	cPCR (blood)	[14,34]
Long-tailed rice mouse	ME	0/11	OM	[29]
(*Oligoryzomys longicaudatus*)	CO	0/1	XD	[30]
	CO	1/2 (50) ^a^	cPCR (blood)	[33]
	CO/VA/ME	8/45 (17.8)	cPCR (blood)	[14,34]
Darwin’s leaf-eared mouse	AP/CO/VA/ME	0/59	OM, XD	[29]
(*Phyllotis darwini*)	CO	1/10 (10.0)	XD, IHA	[30]
	CO	5/62 (8.1)	IHA	[44]
	CO	31/55 (56.0)	cPCR+SB (blood)	[17,31]
	ME	1/4 (25.0)	hnPCR (blood)	[48]
	CO	38/117 (32.5)	cPCR (blood)	[32]
	CO	63/103 (61.2)	cPCR (blood)	[51]
	CO	76/210 (36.2)	cPCR (blood)	[53,54]
	CO	129/379 (34.0) ^a^	cPCR (blood)	[33]
	CO	6/6 (100)	qPCR (blood)	[56]
	CO/VA/ME	73/187 (39.0)	cPCR (blood)	[14,34]
	CO	81/221 (36.7) ^a^	cPCR (blood)	[57]

Abbreviations: Regions AP (Arica y Parinacota), TA (Tarapacá), AN (Antofagasta), AT (Atacama), CO (Coquimbo), VA (Valparaíso), ME (Metropolitana), OH (O’Higgins); NK (not known); OM (optical microscopy); XD (xenodiagnosis); IHA (indirect hemagglutination); cPCR (conventional polymerase chain reaction); cPCR+SB (cPCR and Southern blot); hnPCR (hemi-nested PCR); qPCR (quantitative PCR). ^a^ Detailed data requested to the corresponding author. ^b^ For cumulative datasets the maximum number of the individuals tested is reported. ^c^ Domesticated mammals. Notes: (i) When more than one diagnosis procedure was applied to the same samples, the highest frequency of infection, or the total frequency of infection, considering all the diagnostic tests is reported; (ii) current species names are used [1]; (iii) see the map with the location of Chilean regions in Figure 2; (iv) complete table with tested but negative mammals in Table S1 in Supplementary Materials.

**Table 3:** Alien mammals infected by *Trypanosoma cruzi* in Chile.

ORDER/Species	Region(s)	Positive/ Total Tested (%)	Assay Type (Sample Type)	Reference
ARTIODACTYLA				
Cattle	NK	0/2	XD	[42]
(*Bos taurus*)	ME	0/2	XD	[58]
	CO	0/1	IHA	[16,61,65]
	CO	27/202 (13.4)	IHA	[66]
Goat	AT	0/82	OM, XD	[29]
(*Capra hircus*)	AP/TA/AN/AT/CO/VA/ME/OH	1/233 (0.4) ^a^	XD	[15,42,43]
	CO	0/2	XD	[67]
	NK	0/3	XD	[68]
	CO	32/180 (17.8)	IHA	[69]
	CO	25/265 (9.4) ^a^	IHA	[16,59,61,65]
	AP/TA	0/45^a^	IHA	[16,36,39,65]
	AN	7/98 (7.1) ^a^	IHA	[16,37,39,65]
	AT	7/100 (7.0) ^a^	IHA	[16,60,65]
	VA	0/52 ^a^	IHA	[16,62,65]
	ME	2/11 (18.2) ^a^	IHA	[16,63,65]
	OH	1/26 (3.9) ^a^	IHA	[16,64,65]
	CO	11/316 (3.5)	IHA	[70]
	ME	55/841 (6.5)	IIF	[71]
	CO	21/42 (50.0)	cPCR+SB (blood)	[17,31]
	CO	35/100 (35.0)	IIF, XD-cPCR	[72]
Sheep	AP/TA/AN/AT/CO/VA/ME/OH	0/99	XD	[42]
(*Ovis aries*)	CO	2/42 (4.8) ^a^	IHA	[16,59,61,65]
	AP/TA	7/161 (4.4) ^a^	IHA	[16,36,39,65]
	AN	4/147 (2.7) ^a^	IHA	[16,37,39,65]
	AT	3/16 (18.8) ^a^	IHA	[16,60,65]
	VA	1/33 (3.0) ^a^	IHA	[16,62,65]
	ME	0/1	IHA	[16,63,65]
	OH	4/25 (16.0) ^a^	IHA	[16,64,65]
CARNIVORA				
Dog	AT	16/46 (34.8) ^a^	OM, XD	[73,74,76]
(*Canis lupus familiaris*)	AN/AT/ME	37/184 (20.1)	OM, XD	[75,77]
	ME	2/29 (6.9)	XD	[78]
	AT	2/13 (15.4)	XD	[29]
	ME	24/1026 (2.3)	XD	[79]
	AT	23/104 (22.1)	XD	[80]
	AP/TA/AN/AT/CO/VA/ME/OH	318/3591 (8.9) ^a^	XD	[15,40,41,42,43]
	CO	1/15 (6.7)	XD	[67]
	OH	0/25	XD	[81]
	ME	2/98 (2.0)	XD	[58]
	ME	8/86 (9.3)	XD	[82]
	AN/AT/VA/ME	45/1101 (4.1)	XD	[68]
	CO	44/304 (14.5) ^a^	IHA	[16,59,61,65]
	AP/TA	4/203 (2.0) ^a^	IHA	[16,36,39,65]
	AN	4/65 (6.2) ^a^	IHA, IIF	[16,37,39,65,84]
	AT	8/73 (11.0) ^a^	IHA	[16,60,65,83]
	VA	7/374 (1.9) ^a^	IHA	[16,62,65]
	ME	71/617 (11.5) ^a^	IHA	[16,63,65]
	OH	14/540 (2.6) ^a^	IHA	[16,64,65]
	CO	40/202 (19.8)	IHA	[66]
	CO	20/288 (6.9)	IHA	[70]
	NK	4/36 (11.1)	XD	[16]
	TA	3/29 (10.4)	ELISA	[85]
	VA	8/28 (28.6)	cPCR	[86]
	TA/CO	38/108 (35.2)	nPCR	[87]
	ME	19/111 (17.1) ^b^	Real-time PCR (blood)	[19]
Domestic cat	AT	4/22 (18.2) ^a^	OM, XD	[73,74,76]
(*Felis catus*)	AN/AT/ME	6/136 (4.5) ^a^	OM, XD	[75,77]
	ME	1/8 (12.5)	XD	[78]
	AT	2/8 (25.0)	XD	[29]
	ME	8/595 (1.4)	XD	[79]
	AT	11/47 (23.4)	XD	[80]
	AP/TA/AN/AT/VA/ME/OH	217/1892 (11.5) ^a^	XD	[15,40,41,42,43]
	CO	0/9	XD	[67]
	OH	0/10	XD	[81]
	ME	0/48	XD	[58]
	ME	1/27 (3.3)	XD	[82]
	AN/AT/VA/ME	11/522 (2.1)	XD	[68]
	CO	23/214 (10.8) ^a^	IHA	[16,59,61,65]
	AP/TA	15/140 (10.7) ^a^	IHA	[16,36,39,65]
	AN	1/32 (3.1) ^a^	IHA	[16,37,39,65]
	AT	15/165 (9.1) ^a^	IHA	[16,60,65,83]
	VA	2/197 (1.0) ^a^	IHA	[16,62,65]
	ME	33/304 (10.9) ^a^	IHA	[16,63,65]
	OH	2/93 (2.2) ^a^	IHA	[16,64,65]
	NK	4/19 (21.1)	XD	[16]
LAGOMORPHA				
Rabbit	AP/AT/ME	0/27	OM, XD	[29]
(*Oryctolagus cuniculus*)^c^	AP/TA/AN/AT/CO/VA/ME	2/209 (1.0) ^a^	XD	[15,40,41,42,43]
	CO	0/2	XD	[65]
	CO	18/149 (12.1) ^a^	IHA	[16,59,61,65]
	AP/TA	1/182 (0.6) ^a^	IHA	[16,36,39,65]
	AN	7/145 (4.8) ^a^	IHA	[16,37,39,65]
	AT	0/158 ^a^	IHA	[16,60,65,83]
	VA	0/15 ^a^	IHA	[16,62,65]
	ME	3/47 (6.4) ^a^	IHA	[16,63,65]
	OH	0/8	IHA	[16,64,65]
	CO	22/58 (37.9) ^a^	cPCR, hnPCR	[88]
PERISSODACTYLA				
Ass	AT	0/17	XD	[29]
(*Equus asinus*)	NK	0/74	XD	[42]
	CO	0/1	XD	[67]
	AT	0/21	IHA	[60]
	AP/TA/AN	0/12	IHA	[37,39]
	CO	0/2	IHA	[61]
	VA	0/3	IHA	[62]
	ME	0/1 ^a^	IHA	[16,63,65]
	CO	18/101 (17.8)	IHA	[66]
Horse	NK	0/13	XD	[42]
(*Equus caballus*)	AT	0/10 ^a^	IHA	[16,60,65]
	CO	16/101 (15.8)	IHA	[66]
RODENTIA				
House mouse	AT/ME	0/37	OM, XD	[29]
(*Mus musculus*) ^c^	CO	5/6 (83.3)	qPCR (blood)	[56]
Norway rat	AT/ME	0/4	OM	[29]
(*Rattus norvegicus*) ^c^	CO/VA/ME	5/7 (71.4) ^b^	cPCR (blood)	[14,34]
Black rat	AP/AT/ME	0/11	OM, XD	[29]
(*Rattus rattus*) ^c^	ME	10/44 (22.7)	hnPCR (blood)	[48]
	CO	46/55 (83.6)	qPCR (blood)	[56]
	CO/VA/ME	14/30 (46.7)	cPCR (blood)	[14,34]

Abbreviations: Regions AP (Arica y Parinacota), TA (Tarapacá), AN (Antofagasta), AT (Atacama), CO (Coquimbo), VA (Valparaíso), ME (Metropolitana), OH (O’Higgins); NK (not known); OM (optical microscopy); XD (xenodiagnosis); IHA (indirect hemagglutination), IIF (indirect immunofluorescence), ELISA (enzyme-linked immunosorbent assay), cPCR (conventional polymerase chain reaction), cPCR+SB (cPCR and Southern blot); hnPCR (hemi-nested PCR); qPCR (quantitative PCR). ^a^ For cumulative datasets, the maximum number of individuals tested is reported. ^b^ Some data requested to the corresponding author. ^c^ Free-ranging mammals. Notes: (i) When more than one diagnosis procedure was applied to the same samples, the highest frequency of infection or the total frequency of infection, considering all the diagnostic tests, is reported; (ii) current species names are used [1]; (iii) see the map with the location of Chilean regions in Figure 2; (iv) the complete table with tested but negative mammals in Table S2 in Supplementary Materials.

## Supplementary Materials

The following are available online at https://www.mdpi.com/2076-0817/9/8/661/s1, Table S1: Native mammals tested for *Trypanosoma cruzi* in Chile, Table S2: Alien mammals tested for *Trypanosoma cruzi* in Chile, Table S3: Dataset of the records.
